# Can Chronic Nitric Oxide Inhibition Improve Liver and Renal Dysfunction in Bile Duct Ligated Rats?

**DOI:** 10.1155/2015/298792

**Published:** 2015-11-25

**Authors:** Mona Fouad Mahmoud, Sara Zakaria, Ahmed Fahmy

**Affiliations:** Department of Pharmacology and Toxicology, Faculty of Pharmacy, University of Zagazig, Zagazig 44519, Egypt

## Abstract

The aims of the present work were to study the effects of chronic NO inhibition on liver cirrhosis and to analyze its relationship with liver and kidney damage markers. Two inhibitors of NO synthesis (inducible NO synthase (iNOS) inhibitor, aminoguanidine (AG), and nonselective NOS inhibitor, L-nitroarginine methyl ester (L-NAME)) were administered for 6 weeks to bile duct ligated (BDL) rats 3 days after surgery. The present study showed that BDL was associated with liver injury and renal impairment. BDL increased liver NO content and myeloperoxidase (MPO) activity. This was corroborated by increased oxidative stress, TNF-*α*, TGF-1*β*, and MMP-13 genes overexpression. Although both drugs reduced NO synthesis and TNF-*α* gene overexpression, only AG improved renal dysfunction and liver damage and reduced liver oxidative stress. However, L-NAME exacerbated liver and renal dysfunction. Both drugs failed to modulate TGF-1*β* and MMP-13 genes overexpression. In conclusion, inhibition of NO production by constitutive nitric oxide synthase (cNOS) plays a crucial role in liver injury and renal dysfunction while inhibition of iNOS by AG has beneficial effect. TNF-*α* is not the main cytokine responsible for liver injury in BDL model. Nitric oxide inhibition did not stop the progression of cholestatic liver damage.

## 1. Introduction

Increased nitric oxide (NO) is well recognized in patients with cholestatic liver diseases. Actually high levels of circulating bile salts during cholestasis disrupt intestinal mucosal barrier resulting in translocation of enteric bacteria to the mesenteric lymph nodes and the liver [1] and resulting endotoxemia is responsible for augmented nitric oxide (NO) synthesis by inducible NO synthase (iNOS) [2]. Excessive generation of NO has been observed both in experimental cholestasis and in primary biliary cirrhosis patients. The increase in hepatic and plasma circulating levels of NO and cytokines is the determinant for the hepatocellular injury and the rapid progression of hepatic dysfunction in cholestatic settings [3].

NO has a dual role. Beneficial role is mediated by its circulatory effects and its free radical scavenger properties. Under normal conditions NO has beneficial effect on vascular control including modulation of vascular tone and inflammation. The induction of NO synthesis in abnormal situations allows a more efficient defense not only because of local vascular effects but also because NO is thought to be involved in the macrophage-dependent killing of parasites and possibly cancer cells.

Negative role is mediated through its local toxic effects. NO has a multitude of potentially toxic effects, although many of these are probably mediated by oxidation products rather than by NO itself [4].

Strong inhibition of NO synthesis maintained for long periods by L-NAME may lead to adverse effects through different mechanisms [5]. Previous studies showed that inhibition of NO production in BDL rats may decrease liver blood flow promoting clot formation [6]. It also favors the production of oxyradicals [7]. Selective inhibition of iNOS by chronic administration of aminoguanidine (AG) could reduce systemic NO levels as it suppresses iNOS expression and activity in aorta of BDL rats. It also improves liver function possibly because of its ability to increase hepatic cNOS activity and to correct the systemic hemodynamic disorders by decreasing vascular NO production [8].

The role of NO produced by iNOS in bile duct ligation model was previously investigated. Some studies showed that iNOS gene transfer could inhibit hepatocytes apoptosis [9]. Furthermore, Dirlik et al. [10] reported that BDL caused a significant increase in iNOS staining on the 3rd day following surgery and decreased on the 5th day after BDL and reduction of iNOS expression was associated with increased hepatocytes apoptosis. However, the changes in NO production after longer periods following BDL have not been yet investigated.

From this point of view, the aim of the present work was to study the role of complete and partial inhibition of nitric oxide synthase enzymes in liver fibrosis and renal dysfunction induced by bile duct ligation in rats after 6 weeks of BDL. In addition the correlation between NO inhibition and matrix formation, and growth factors and the inflammatory process mediating liver fibrosis was also investigated.

## 2. Materials and Methods

### 2.1. Animals

Adult male Wistar rats weighing 180 ± 220 g were used in the present investigation. The animals were obtained from National Research Center, Cairo, Egypt. They were kept under constant environmental and nutritional conditions throughout the period of study. They were housed as six rats per cage in plastic cages with wood shave bedding and fed normal standard diet. They had free access to water and food. The protocol of the present study was approved by the Animal Care and Use Committee of the Pharmacology Department, Faculty of Pharmacy, Zagazig University, Egypt. Every effort was done to minimize the number of animals and their suffering.

### 2.2. Materials

Aminoguanidine (AG) and L-NAME were supplied from Sigma Co., USA. The required dose of AG was dissolved in normal saline but the required dose of L-NAME was dissolved in drinking water. All drugs and vehicle were given to rats by oral gavage.

### 2.3. Experimental Design

The animals were randomly divided into 5 groups of 20 rats/per each group. Group (1) received normal saline (2 mL/kg, orally) for 6 weeks and represents normal control group. Group (2) underwent a midline incision and manipulation of the bile duct without ligation and received saline (2 mL/kg, orally) and served as sham group. Group (3) was bile duct ligated and received saline (2 mL/kg, orally) and served as BDL group. Group (4) was bile duct ligated and received aminoguanidine (50 mg/kg, orally). Group (5) was bile duct ligated and received L-NAME (2 mg/kg, orally).

All treatments were given starting from 3rd day after surgery and for 6 weeks as single daily dose.

### 2.4. Induction of Fibrosis

Fibrosis was induced in rats by the ligation of the common bile duct. Bile duct ligation was performed under general anesthesia with a mixture of ketamine hydrochloride (50 mg/kg) and diazepam (3 mg/kg) [11]. The common bile duct was manipulated and then doubly ligated with 4-0 silk threads and excised between the ligatures to prevent regeneration. In sham operated group, the bile duct was identified, manipulated, and left* in situ* without ligation. Meloxicam in a dose of 1 mg/kg [12] IM was given for 3 days after surgery to reduce pain. Meloxicam has a good record for effectiveness and safety for both short-term and long-term use in animals. Rats were also injected with Penicillin G (aqua-pen vial) by deep IM injection [13] for 3 days after surgery for prophylaxis against infection.

### 2.5. Blood Sampling and Serum Preparation

At the end of the experiment, blood was collected from the orbital sinus of rats [14] in clean dry centrifuge tubes. For the preparation of serum, blood was collected into tubes without anticoagulant. The tubes were left to clot for 15 minutes at room temperature. Serum was separated after centrifugation at 3500 r.p.m. (10,000 ×g) for 15 minutes using Heraeus Sepatech centrifuge (Labofuge 200, DJB Labcare Company). Serum was divided into two aliquots and all were frozen. The first aliquot was used for the determination of alanine aminotransferase (ALT), aspartate aminotransferase (AST), lactate dehydrogenase (LDH), and total bilirubin. The second aliquot was used for determination of creatinine and urea.

### 2.6. Tissue Sampling

Animals were anaesthetized by ether and livers were perfused with phosphate buffered solution (PBS) containing 0.16 mg/mL heparin. Livers were isolated and dissected into 3 parts. All parts were immersed immediately in liquid nitrogen and kept at −80°C. These parts were used to measure tumor necrosis factor alpha (TNF-*α*), transforming growth factor one beta (TGF-1*β*), matrix metalloproteinase-13 (MMP-13), myeloperoxidase (MPO), malondialdehyde (MDA), reduced glutathione content (GSH), and nitric oxide (NO).

### 2.7. Biochemical Analysis

#### 2.7.1. Liver Function Tests

Serum ALT and AST activities were determined by a colorimetric method according to the principle of Reitman and Frankel [15].

#### 2.7.2. Liver Cell Death

Serum lactate dehydrogenase activity as indicator of cytolytic cell death was determined using a kinetic method according to the method of Fasce Jr. and Rej [16].

#### 2.7.3. Serum Total Bilirubin

It was measured colorimetrically as described by Gellis et al. [17].

#### 2.7.4. Kidney Function Tests

Serum urea was measured colorimetrically as described by Fawcett and Scott [18] and also creatinine level was measured colorimetrically as described by Bowers and Wong [19].

#### 2.7.5. Liver TNF-*α*, TGF-1*β*, and MMP-13

Detection of TNF-*α*, TGF-1*β*, and MMP-13 gene expression was performed by semiquantitative reverse transcriptase-polymerase chain reaction (RT-PCR).

Briefly, total RNA was extracted from liver tissue using E.Z.N.A. RNA extraction kit. The extracted RNA was reverse transcribed into cDNA using RT-PCR kit (Stratagene, USA). The cDNA was amplified using the following primers:

 TNF alpha, Forward: 5′-ATTGGCAAATGGGAAAATGA-3′
 Reverse: 5′-TTATGACCTCCTTTTGGTCTGA-3′,
 TGF beta, Forward: 5′-TTGAGTGTCAGCCCACAGAG-3′
 Reverse: 5′-TCCGACAGCCACACTTCTTC-3′,
 MMP-13, Forward: 5′-GCTGGTCAGTCGCCCTTTT-3′
 Reverse: 5′-GCTAAGGAAAGCAGAGAGGGATT-3′.
 Gene expression of *β*-actin was used as a house keeping gene.

The primer sequence of *β*-actin was Forward: 5′-TCA CCC TGA AGT ACC CCA TGG AG-3′ and Reverse: 5′-TTG GCC TTG GGG TTC AGG GGG-3′. At the end of the amplification process, the DNA product was detected using agarose gel electrophoresis. Semiquantitation was performed using the gel documentation system (BioDO, Analyser) supplied by Biometra. Relative expression of each studied gene (*R*) was calculated following the formula: *R* = Densitometrical Units of each studied gene/Densitometrical Units of *β*-actin.

#### 2.7.6. Liver MPO

Liver MPO activity was determined by a colorimetric method using myeloperoxidase chlorination activity determination kit as described by Kettle and Winterbourn [20].

#### 2.7.7. Liver GSH and Membrane Lipid Peroxidation

The reduced form of glutathione (GSH) was determined in the liver homogenate by colorimetric method according to Beutler et al. [21]. Malondialdehyde (MDA) content as indicator of lipid peroxidation was determined in the liver homogenate, by a colorimetric method according to Ohkawa et al. [22].

#### 2.7.8. Liver Nitric Oxide

Liver content of nitric oxide was quantitatively measured indirectly as nitrite and nitrate by a colorimetric method as described by Montgomery and Dymock [23] using a diagnostic kit supplied by Tocris Bioscience, Boston Biochem join R&D systems (Minneapolis, USA).

### 2.8. Statistical Analysis

Results were expressed as mean ± S.E.M. Graph Pad Prism software version 5 was used to perform statistical analysis. Comparison between different groups was carried out using one-way analysis of variance (ANOVA) followed by Tukey's* post hoc* test. The statistical associations between functional parameters were assayed using Spearman nonparametric correlation analysis. *P* values less than 0.05 were considered significant.

## 3. Results

### 3.1. General Observations

The animals started to have the signs of cholestasis such as jaundice, dark urine, and steatorrhea at the 4th day from the bile duct ligation operation with higher mortality rate during the first 2 weeks after the operation; then mortality rate decreased during the 3rd and 4th weeks and then increased at last two weeks. Untreated BDL group had the highest mortality rate.

### 3.2. Effect on Liver Enzymes and Total Bilirubin

Table 1 showed that BDL significantly increased serum activities of ALT, AST, and serum total bilirubin level as compared to sham group (*P* < 0.05). Aminoguanidine caused significant (*P* < 0.05) reduction of ALT, AST serum activities and serum total bilirubin levels. However, L-NAME caused significant (*P* < 0.05) increase in the activities of serum ALT, AST, and total bilirubin level.

### 3.3. Effect on Liver Cell Death

Bile duct ligation significantly increased serum activity of LDH, an indicator of necrotic cell death as compared to sham group (*P* < 0.05). Aminoguanidine reduced LDH activity as compared to BDL group (Table 1). On the other hand, L-NAME did not affect LDH activity when compared to BDL group (*P* > 0.05).

### 3.4. Effect on Kidney Function

Bile duct ligation significantly increased serum levels of urea and creatinine as compared to sham (*P* < 0.05). Aminoguanidine caused significant (*P* < 0.05) reduction of urea and creatinine as compared to BDL group. L-NAME caused significant (*P* < 0.05) increase in serum urea levels and had no effect on serum creatinine levels as compared to BDL group.

### 3.5. Effect on Oxidative Stress and Nitric Oxide Levels

Bile duct ligation significantly increased liver MDA, a lipid peroxidation product (*P* < 0.05), and significantly decreased liver GSH as compared to sham (*P* < 0.05). Aminoguanidine caused significant (*P* < 0.05) decrease in liver MDA and significant (*P* < 0.05) increase in liver GSH as compared to BDL group (Table 2). L-NAME had no significant effect on liver contents of both MDA and GSH as compared to BDL group (Table 2). Both aminoguanidine and L-NAME caused a significant (*P* < 0.05) reduction of liver NO compared to BDL group (Table 2).

### 3.6. Effect on Inflammation Markers

**Figure 1:**
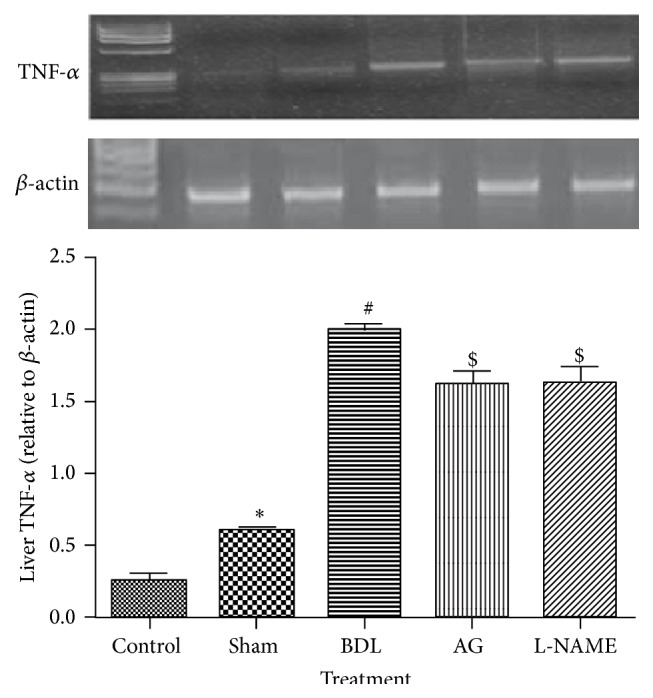
Effect of aminoguanidine (AG) and L-NAME on liver TNF-*α* gene expression relative to *β*-actin in bile duct ligated (BDL) rats and an agarose gel electrophoresis show PCR products of TNF alpha. Lane M: DNA marker with 100 bp; Lane 1: PCR products of TNF alpha in control group; Lane 2: PCR products of TNF alpha in sham group; Lane 3: PCR products of TNF alpha in BDL group; Lane 4: PCR products of TNF alpha in aminoguanidine group; Lane 5: PCR products of TNF alpha in L-NAME group.

Myeloperoxidase is an enzyme released by infiltrating inflammatory cells during inflammation and was increased significantly in BDL rats as compared to sham rats (*P* < 0.05). Aminoguanidine and L-NAME did not affect liver MPO activity (Table 2). TNF-*α* gene expression was upregulated in the liver cells of BDL when compared to sham group (*P* < 0.05). Both aminoguanidine and L-NAME produced significant reduction of TNF-*α* gene expression (*P* < 0.05) as shown in Figure 1.

### 3.7. Effect on Growth Factors and Matrix Formation

**Figure 2:**
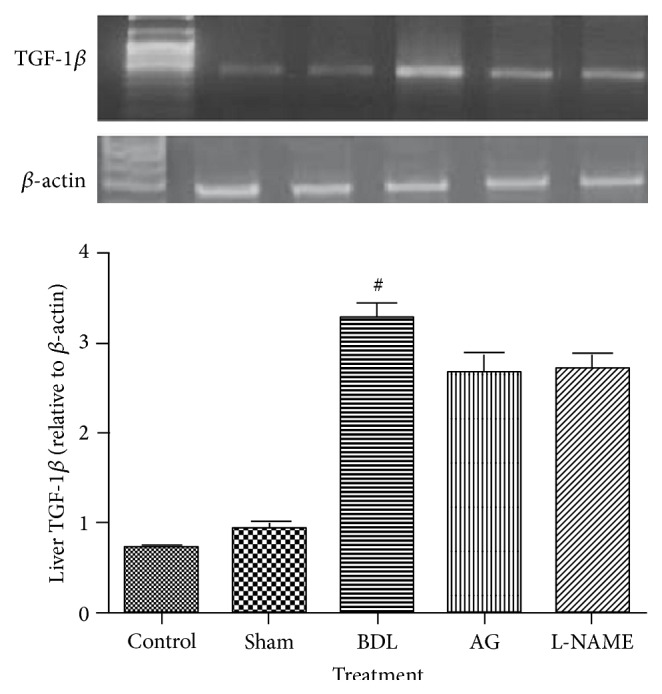
Effect of aminoguanidine (AG) and L-NAME on liver TGF-1*β* gene expression relative to *β*-actin in bile duct ligated (BDL) rats and an agarose gel electrophoresis show PCR products of TGF beta in different studied groups. Lane M: DNA marker with 100 bp; Lane 1: PCR products of TGF beta in control group; Lane 2: PCR products of TGF beta in sham group; Lane 3: PCR products of TGF beta in BDL group; Lane 4: PCR products of TGF beta in aminoguanidine group; Lane 5: PCR products of TGF beta in L-NAME group.

**Figure 3:**
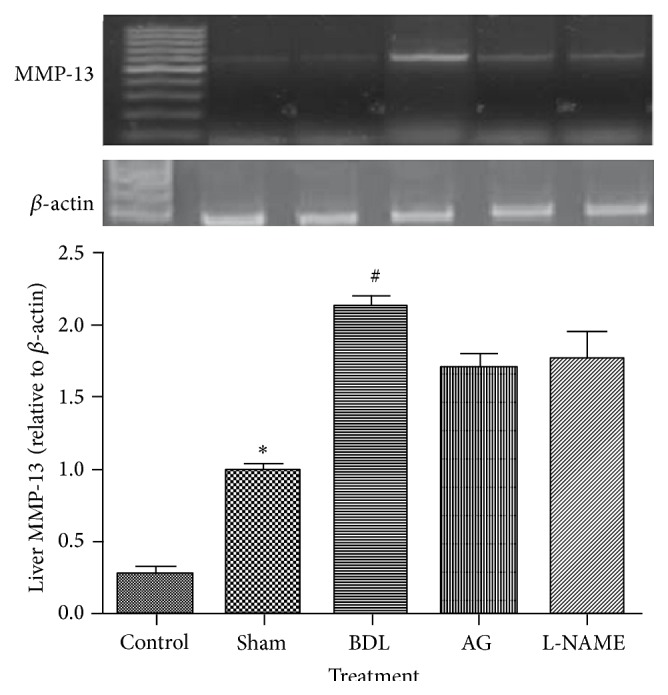
Effect of aminoguanidine (AG) and L-NAME on liver MMP-13 gene expression relative to *β*-actin in bile duct ligated (BDL) rats and an agarose gel electrophoresis show PCR products of MMP-13 in different studied groups. Lane M: DNA marker with 100 bp; Lane 1: PCR products of MMP-13 in control group; Lane 2: PCR products of MMP-13 in sham group; Lane 3: PCR products of MMP-13 in BDL group; Lane 4: PCR products of MMP-13 in aminoguanidine group; Lane 5: PCR products of MMP-13 in L-NAME group.

Bile duct ligation induced a significant increase in liver expression of both TGF-1*β* and MMP-13 as compared to sham rats. Aminoguanidine and L-NAME had no significant effect on both TGF-1*β* and MMP-13 gene expression as compared to BDL group (Figures 2 and 3).

### 3.8. Correlation Analysis between Liver Content of Nitrite and Different Studied Parameters

There was positive correlation between liver nitrite and serum activity of LDH (*r* = 0.4 at *P* < 0.05), serum creatinine level (*r* = 0.3 at *P* < 0.05), and liver TGF-1*β*, MMP-13, TNF-*α*, MDA, and GSH (*r* = 0.4 for all at *P* < 0.05). However, serum activities of ALT, AST, and total bilirubin level were not correlated to liver nitrite (*r* = 0.2, 0.1, and 0.1, resp., at *P* > 0.05). Serum urea level and liver MPO activity were not correlated to liver nitrite (*r* = 0.1 and 0.3, resp., at *P* > 0.05).

## 4. Discussion

Although some previous studies investigated the effect of aminoguanidine on cholestatic liver damage, the present study was the first up to our knowledge which compares chronic partial or complete inhibition of NO by aminoguanidine or L-NAME, respectively, on liver and renal dysfunction in bile duct ligated rat model. It is one of the longest treatment periods in the literature using these inhibitors. We found that bile duct ligation for six weeks caused a significant increase in serum activities of ALT, AST and in serum level of total bilirubin compared to sham group indicating liver injury due to cholestasis. The mortality rate in the untreated bile duct rats was the highest rate among the experimental groups. This may be attributed to the great elevation of bilirubin in the blood of untreated animals during the first two weeks after surgery and due to complications of hepatic damage at the last two weeks of the experiment. It also increased serum levels of urea and creatinine as markers for kidney function compared to sham group. Lactate dehydrogenase, a marker of cytolytic cell death, was also elevated. Liver genes expression of TGF-1*β*, TNF-*α*, and MMP-13, MPO activity, MDA, and nitric oxide levels were significantly increased but the liver content of GSH was decreased compared to sham group.

One of the proposed mechanisms by which BDL induces liver injury was investigated in the present study, the role of NO in liver injury. Some studies suggested that NO has a dual role in cholestatic liver disease; one is beneficial, mediated by its circulatory effects, and the second is negative, through its local toxic effects [24]. Increased NO level and NO synthase activity in patients with liver cirrhosis have adverse effects on the functions of renal tubules and glomeruli [25]. Inhibition of NO synthase prevented the development of renal failure in an animal model of hepatorenal syndrome [26].

Based on the previous studies that indicate that nitric oxide (NO) plays an important role in the systemic and renal alterations of liver cirrhosis, in this study, we used aminoguanidine (AG), a preferential inhibitor of inducible nitric oxide synthase (iNOS), to evaluate the role of this NOS isoform in the pathogenesis of liver cirrhosis and its subsequent alteration in renal function. AG was orally administered in a dose 50 mg/kg as it totally inhibits iNOS with no effect on constitutive NOS but the 100 mg/kg dose also inhibits some constitutive NOS isoform [27].

Oral administration of AG (50 mg/kg) for bile duct ligated rats three days after surgery for six weeks produced a significant decrease in the serum activities of ALT, AST, LDH, and serum total bilirubin level compared to BDL group. It also decreased liver content of TNF-*α* compared to BDL group. Previous studies showed that AG may be useful in the preservation of liver injury in cholestasis as it reduces cytokine induced liver damage and ductular proliferation [28]. Inhibition of iNOS is thought to control the injury of liver. The present study showed that AG decreased liver NO production compared to BDL group. It was reported previously that iNOS was expressed in liver tissue from control rats but its expression was increased in BDL animals [24]. On the other hand, cNOS was expressed to a similar extent in normal and BDL animals which make the iNOS responsible for liver injury.

AG improved renal dysfunction as revealed by reduction of both urea and creatinine levels. Previous studies showed that iNOS is involved in the renal alterations observed in BDL animals [29]. NO plays an important role as a mediator of the systemic and renal alterations of liver cirrhosis. NO inhibition in experimental animals resulted in a reduction of the hyperdynamic circulation [30] and produces the beneficial effects on renal excretory function [31].

The present study showed that aminoguanidine has antioxidant effects. It decreased liver MDA and increased liver GSH compared to BDL group. During cholestasis, there is a high oxidative stress and more free radicals are produced. Interactions between reactive oxygen species and reactive nitrogen species, mainly NO, could mediate some of the pathological effects associated with chronic inflammation [32]. AG may attenuate liver and kidney damage during cholestasis through its inhibition of iNOS and its antioxidant effect.

Although aminoguanidine improved liver and renal dysfunction, it failed to modulate the fibrotic process in liver. The present study showed that AG did not modulate TGF-1*β* or MMP-13 genes expression. In spite of reducing liver TNF-*α* gene expression, AG had no significant effect on MPO activity indicating that it could not modulate leukocyte infiltration. It seems that TNF-*α* is not the only player in the fibrotic process in liver. Other cytokines may have a more important role in fibrosis induced by BDL. A previous study of Bautista-García et al. [33] showed that the inhibition of iNOS by 0.1% AG reduced TGF-1*β* over expression in 5/6 nephrectomized rats. Another study of Kelly et al. [34] revealed that AG (1 g/L) reduces TGF-1*β* in diabetic nephropathy due to inhibition of advanced glycation end products (AGEs) formation but not due to NO inhibition. These variations in results may be related to the difference in experimental model and AG dose.

In contrast to the present findings, AG increased the expression of MMP-13 in diabetic mice kidney [35] via blocking AGEs formation. It was also reported that both AG and L-NAME upregulated MMP-13 mRNA expression in rat thioacetamide-induced fibrosis model [36]. The AG effects were more pronounced and it also inhibits TGF-1*β*. Both NOS inhibitors developed a clear profibrotic effect in the liver. Aminoguanidine was more fibrotic than L-NAME. Again in these studies, AG was administered in a dose of 100 mg/kg for three months, which may inhibit both constitutive and inducible NOS.

In this study, we also used L-NAME, a potent nonselective NOS inhibitor to determine the effects of nonselective NO inhibition during cholestatic liver disease. Oral administration of L-NAME (2 mg/kg) for BDL rats three days after surgery for six weeks produced a significant increase in the activities of serum enzymes of ALT, AST and serum level of total bilirubin with no effect on serum LDH compared to BDL group, indicating an aggravation of liver dysfunction and damage. It had no effect on liver contents of TGF-1*β*, MMP-13, and MPO but it decreased the liver content of NO compared with BDL group. Previous studies showed similar effects on liver [24]. We showed for the first time that L-NAME administration has detrimental effect on the renal function as manifested by elevation of urea level when compared by untreated BDL rats.

The deleterious effect of L-NAME may be attributed to inhibition of NO production by cNOS which may decrease liver blood flow, promoting clot formation and ROS generation [7].

## 5. Conclusion

Both aminoguanidine and L-NAME did not modulate the fibrotic process in liver of BDL rats in spite of reducing liver TNF-*α* gene expression. AG only decreased liver and kidney dysfunction due to its partial inhibition of nitric oxide synthase and antioxidant effects. L-NAME increased liver injury and deteriorates renal function due to its complete inhibition of nitric oxide synthase.

## Figures and Tables

**Table 1:** Effect of different treatments on liver and renal functions of bile duct ligated rats.

Treatment	Control	Sham	BDL	Aminoguanidine	L-NAME
ALT	24.3 ± 1.8	56.1^*∗*^ ± 2.1	1012^#^ ± 6.4	290.2^$^ ± 3.5	1517^$^ ± 8.3
AST	126.3 ± 5.9	310.4^*∗*^ ± 5.7	1379^#^ ± 11.0	1102^$^ ± 5.3	1801^$^ ± 6.4
LDH	929.7 ± 6.9	1637^*∗*^ ± 14.7	2607^#^ ± 10.1	2050^$^ ± 18.8	2428^$^ ± 16.1
T. bilirubin	0.02 ± 0.002	0.08 ± 0.003	4.06^#^ ± 0.1	3.51^$^ ± 0.04	6.07^$^ ± 0.07
Urea	11.8 ± 0.4	31.5^*∗*^ ± 0.8	327.7^#^ ± 3.8	172.4^$^ ± 2.7	516.4^$^ ± 5.8
Creatinine	0.24 ± 0.02	0.72^*∗*^ ± 0.05	2.72^#^ ± 0.06	1.95^$^ ± 0.06	2.78 ± 0.02

Data are presented as mean ± S.E., *n* = 6. ^*∗*^Significantly different from control at *P* < 0.05^*∗*^, ^#^significantly different from sham at *P* < 0.05, and ^$^significantly different from BDL at *P* < 0.05, using one-way ANOVA and Tukey's *post hoc* test.

**Table 2:** Effect of different treatments on nitrosative and oxidative stress in bile duct ligated rats.

Treatment	Control	Sham	BDL	Aminoguanidine	L-NAME
MPO	1.8 ± 0.1	2.9^*∗*^ ± 0.2	4.6^#^ ± 0.2	4.2 ± 0.3	4.5 ± 0.3
GSH	2.7 ± 0.2	1.6^*∗*^ ± 0.1	0.4^#^ ± 0.1	0.9^$^ ± 0.1	0.6 ± 0.1
MDA	24.6 ± 0.1	41.1^*∗*^ ± 1.3	62.7^#^ ± 2.3	52.7^$^ ± 2.6	58.2 ± 2.5
NO	0.21 ± 0.02	0.33^*∗*^ ± 0.03	0.94^#^ ± 0.02	0.19^$^ ± 0.02	0.08^$^ ± 0.004

Data are presented as mean ± S.E., *n* = 6.  ^*∗*^Significantly different from control at *P* < 0.05,  ^#^significantly different from sham at *P* < 0.05, and ^$^significantly different from BDL at *P* < 0.05, using one-way ANOVA and Tukey's *post hoc* test.

## Abbreviations

AG: Aminoguanidine
BDL: Bile duct ligation/bile duct ligated
L-NAME: L-Nitroarginine methyl ester
MMP-13: Matrix metalloproteinase-13
TNF-*α*: Tumour necrosis factor alpha
TGF-1*β*: Transforming growth factor 1*β*
MPO: Myeloperoxidase
cNOS: Constitutive nitric oxide synthase
iNOS: Inducible nitric oxide synthase.
